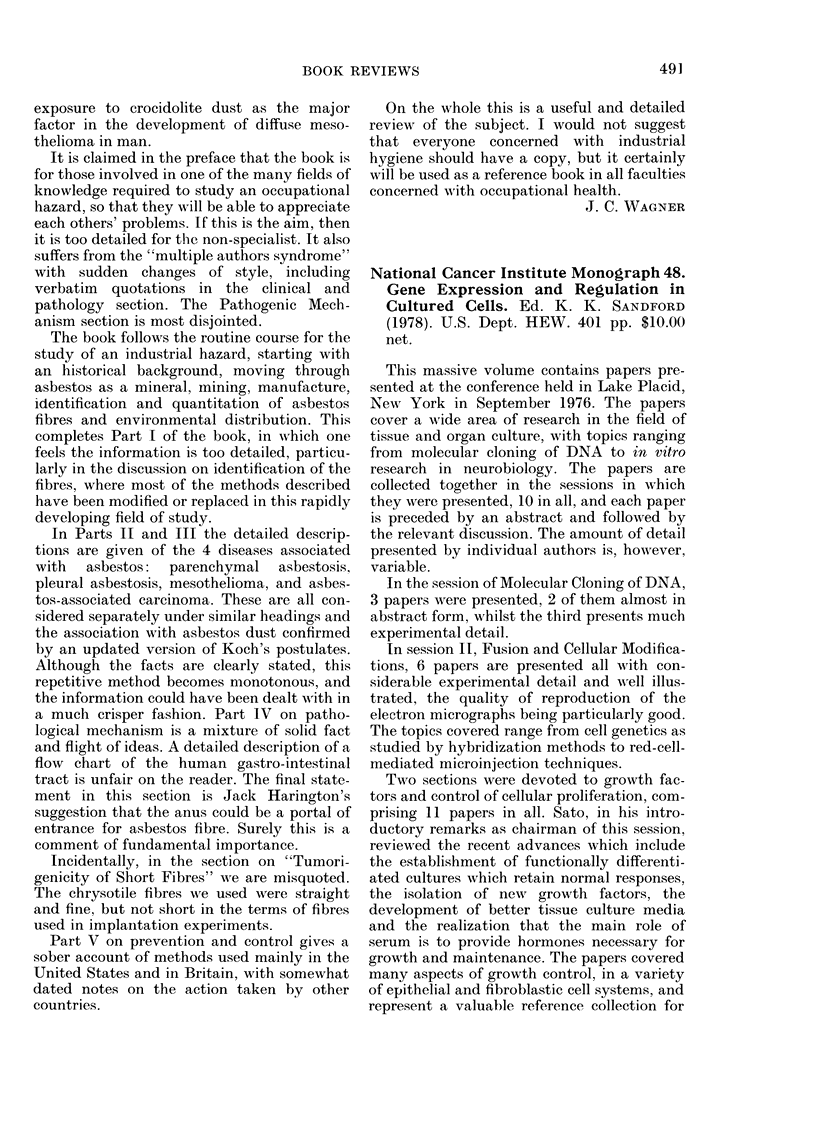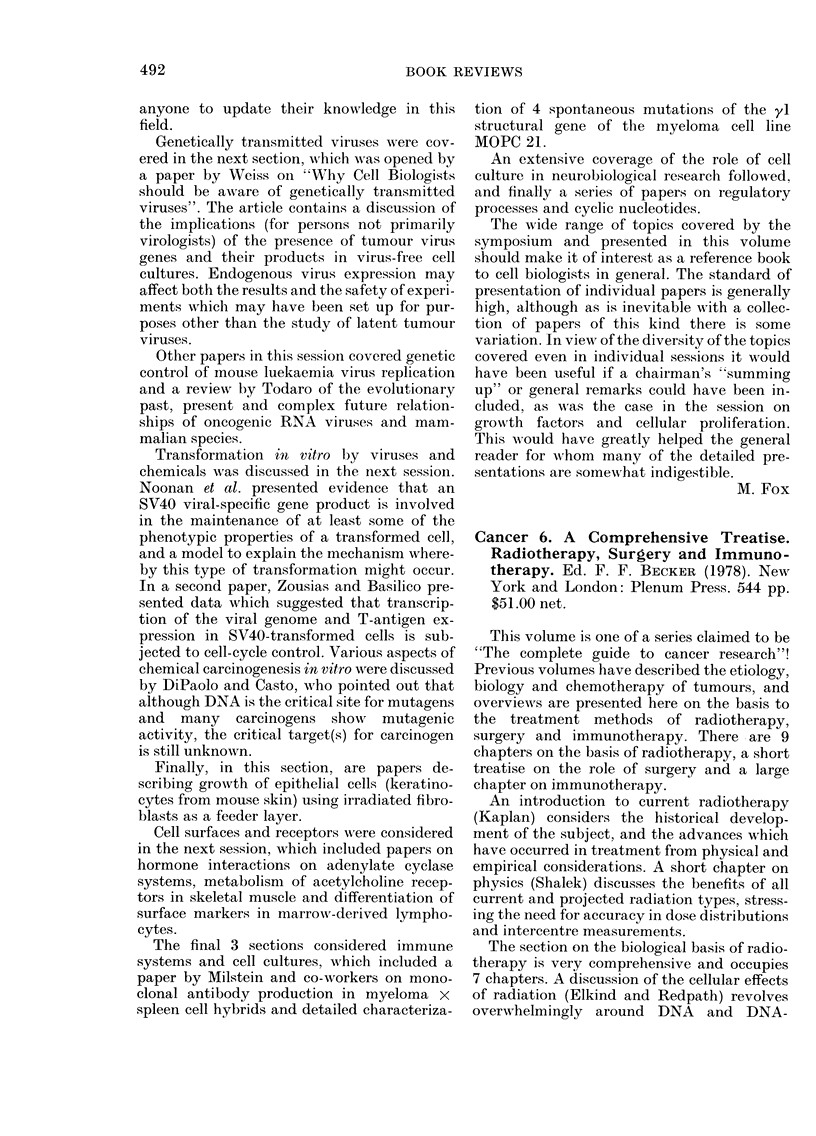# National Cancer Institute Monograph 48. Gene Expression and Regulation in Cultured Cells

**Published:** 1979-04

**Authors:** M. Fox


**National Cancer Institute Monograph 48. Gene Expression and Regulation in Cultured Cells.** Ed. K. K. SANDFORD (1978). U.S. Dept. HEW. 401 pp. $10.00 net.

This massive volume contains papers presented at the conference held in Lake Placid, New York in September 1976. The papers cover a wide area of research in the field of tissue and organ culture, with topics ranging from molecular cloning of DNA to *in vitro* research in neurobiology. The papers are collected together in the sessions in which they were presented, 10 in all, and each paper is preceded by an abstract and followed by the relevant discussion. The amount of detail presented by individual authors is, however, variable.

In the session of Molecular Cloning of DNA, 3 papers were presented, 2 of them almost in abstract form, whilst the third presents much experimental detail.

In session II, Fusion and Cellular Modifications, 6 papers are presented all with considerable experimental detail and well illustrated, the quality of reproduction of the electron micrographs being particularly good. The topics covered range from cell genetics as studied by hybridization methods to red-cell-mediated microinjection techniques.

Two sections were devoted to growth factors and control of cellular proliferation, comprising 11 papers in all. Sato, in his introductory remarks as chairman of this session, reviewed the recent advances which include the establishment of functionally differentiated cultures which retain normal responses, the isolation of new growth factors, the development of better tissue culture media and the realization that the main role of serum is to provide hormones necessary for growth and maintenance. The papers covered many aspects of growth control, in a variety of epithelial and fibroblastic cell systems, and represent a valuable reference collection for anyone to update their knowledge in this field.

Genetically transmitted viruses were covered in the next section, which was opened by a paper by Weiss on "Why Cell Biologists should be aware of genetically transmitted viruses". The article contains a discussion of the implications (for persons not primarily virologists) of the presence of tumour virus genes and their products in virus-free cell cultures. Endogenous virus expression may affect both the results and the safety of experiments which may have been set up for purposes other than the study of latent tumour viruses.

Other papers in this session covered genetic control of mouse luekaemia virus replication and a review by Todaro of the evolutionary past, present and complex future relationships of oncogenic RNA viruses and mammalian species.

Transformation *in vitro* by viruses and chemicals was discussed in the next session. Noonan *et al.* presented evidence that an SV40 viral-specific gene product is involved in the maintenance of at least some of the phenotypic properties of a transformed cell, and a model to explain the mechanism whereby this type of transformation might occur. In a second paper, Zousias and Basilico presented data which suggested that transcription of the viral genome and T-antigen expression in SV40-transformed cells is subjected to cell-cycle control. Various aspects of chemical carcinogenesis *in vitro* were discussed by DiPaolo and Casto, who pointed out that although DNA is the critical site for mutagens and many carcinogens show mutagenic activity, the critical target(s) for carcinogen is still unknown.

Finally, in this section, are papers describing growth of epithelial cells (keratinocytes from mouse skin) using irradiated fibroblasts as a feeder layer.

Cell surfaces and receptors were considered in the next session, which included papers on hormone interactions on adenylate cyclase systems, metabolism of acetylcholine receptors in skeletal muscle and differentiation of surface markers in marrow-derived lymphocytes.

The final 3 sections considered immune systems and cell cultures, which included a paper by Milstein and co-workers on monoclonal antibody production in myeloma $\times$ spleen cell hybrids and detailed characterization of 4 spontaneous mutations of the $\gamma 1$ structural gene of the myeloma cell line MOPC 21.

An extensive coverage of the role of cell culture in neurobiological research followed, and finally a series of papers on regulatory processes and cyclic nucleotides.

The wide range of topics covered by the symposium and presented in this volume should make it of interest as a reference book to cell biologists in general. The standard of presentation of individual papers is generally high, although as is inevitable with a collection of papers of this kind there is some variation. In view of the diversity of the topics covered even in individual sessions it would have been useful if a chairman's "summing up" or general remarks could have been included, as was the case in the session on growth factors and cellular proliferation. This would have greatly helped the general reader for whom many of the detailed presentations are somewhat indigestible.

M. Fox